# Association between atherogenic dyslipidemia and DBS-derived p-tau217 in older adults: evidence from SHARE DBS data

**DOI:** 10.3389/fnagi.2026.1948518

**Published:** 2026-09-22

**Authors:** Zunwei Wang, Yanyan Liu, Xiaofei Sun, Yitong Ling

**Affiliations:** 1 Department of Neurology, The Affiliated Hospital of Qingdao University, Qingdao, China; 2 Qingdao Medical College, Qingdao University, Qingdao, China; 3 Department of Gastroenterology, The Affiliated Hospital of Qingdao University, Qingdao, China

**Keywords:** Alzheimer’s disease, atherogenic index of plasma, dried blood spot, dyslipidemia, phosphorylated tau 217, SHARE

## Abstract

**Background:**

Atherogenic dyslipidemia has been associated with adverse cognitive and dementia-related outcomes, but its relationship with Alzheimer’s disease-related blood biomarkers remains unclear.

**Objective:**

To examine whether the atherogenic index of plasma (AIP) is associated with dried blood spot (DBS)-derived phosphorylated tau 217 (p-tau217) in older adults from Germany, Sweden, and Denmark.

**Materials and methods:**

This cross-sectional analysis included adults aged ≥50 years from Germany, Sweden, and Denmark in the Survey of Health, Ageing and Retirement in Europe DBS biomarker substudy. AIP was calculated as log10(triglycerides/high-density lipoprotein cholesterol). Multivariable linear regression with robust standard errors evaluated standardized natural log-transformed p-tau217, with additional dose–response analyses, comparisons across neurological biomarkers, and sensitivity analyses.

**Results:**

Among 2,676 participants, a 1-SD higher AIP was associated with a 0.074-SD higher ln(p-tau217) (95% CI, 0.034–0.115; *p* < 0.001), corresponding after back-transformation to a 5.2% higher adjusted geometric mean DBS-derived p-tau217 concentration (95% CI, 2.3–8.1%). The highest versus lowest AIP quartile was associated with higher p-tau217 (β, 0.162; 95% CI, 0.052–0.272; *p* = 0.004), although estimates across quartiles were not strictly monotonic. Associations were statistically supported for p-tau217 and total tau, but not for GFAP or NfL after FDR correction. Results were robust in sensitivity analyses.

**Conclusion:**

In this cross-sectional analysis, higher AIP, reflecting atherogenic dyslipidemia, was associated with a modestly higher DBS-derived p-tau217 concentration in older adults. The biological and clinical significance of this association remains to be established in longitudinal and multimodal studies.

## 1 Introduction

Lipid dysregulation is a major driver of atherosclerotic cardiovascular risk. Beyond its vascular consequences, an atherogenic metabolic milieu in later life may be linked to cognitive decline and dementia risk (Mechanick et al., 2020; Livingston et al., 2024). Lipid and glucose metabolic disturbances often coexist with hypertension, obesity, and chronic low-grade inflammation. Experimental evidence further links inflammatory signaling to atherosclerotic plaque vulnerability and stability (Chen et al., 2023). These processes have been associated with vascular injury and Alzheimer’s disease (AD) and related dementias, with proposed links involving cerebral small-vessel disease, blood–brain barrier disruption, and neuroinflammation (Snyder et al., 2015; Nation et al., 2019).

Evidence linking conventional individual lipid measures to cognitive or dementia outcomes has been inconsistent (Iwagami et al., 2021; Af Geijerstam et al., 2025; Anstey et al., 2008). In previous studies, total cholesterol (TC), low-density lipoprotein cholesterol (LDL-C), high-density lipoprotein cholesterol (HDL-C), and triglycerides (TG) have shown varying directions and strengths of association with dementia risk (Iwagami et al., 2021; Af Geijerstam et al., 2025). A lipid phenotype that captures atherogenic dyslipidemia more broadly may therefore capture complementary information beyond individual lipid measures for evaluating the relationship between cardiometabolic risk and brain health.

The atherogenic index of plasma (AIP), defined as log10(TG/HDL-C), captures the lipid pattern characterized by elevated TG and reduced HDL-C and is related to lipoprotein particle size and cholesterol esterification (Dobiásová and Frohlich, 2001). AIP has been associated with vascular events and may provide complementary information beyond individual lipid measures (Onat et al., 2010; Assempoor et al., 2025; Qu et al., 2024; Liu et al., 2024). More recent observational studies have linked higher AIP to poorer cognitive performance and an increased risk of incident dementia (Zhang et al., 2025; Lin et al., 2025). However, these studies have relied primarily on clinical diagnoses or cognitive test scores rather than AD-related molecular biomarkers. Determining whether atherogenic dyslipidemia is associated with AD-related blood biomarkers may provide insight into the relationship between cardiometabolic lipid disturbances and AD-related molecular changes.

Among circulating tau biomarkers, p-tau217 is closely associated with AD-related amyloid and tau pathology, as reflected by amyloid PET, tau PET, and CSF biomarkers (Palmqvist et al., 2020; Ashton et al., 2024), whereas conventional blood total tau is less pathologically specific and may predominantly reflect peripheral rather than brain-derived tau (Gonzalez-Ortiz et al., 2023). The MSD S-PLEX p-tau217 assay used in the present study has shown favorable analytical and diagnostic performance in plasma, while capillary dried-sample approaches using other collection and assay platforms have demonstrated the feasibility of measuring p-tau217 outside conventional venous plasma sampling (Kivisäkk et al., 2024; Huber et al., 2026). To our knowledge, no population-based study has directly examined the association between AIP and DBS-derived p-tau217. Survey of Health, Ageing and Retirement in Europe (SHARE) Wave 6 includes dried blood spot (DBS) biomarker measurements in older European adults, providing a useful setting to investigate whether atherogenic lipid phenotypes are associated with AD-related blood biomarkers (Börsch-Supan et al., 2013; SHARE-ERIC, 2024; SHARE-ERIC, 2026; Börsch-Supan et al., 2024).

Using these data, we examined the association between AIP and DBS-derived blood p-tau217 levels among adults aged 50 years or older from Germany, Sweden, and Denmark. We further characterized dose–response and potential nonlinear patterns, compared AIP with conventional lipid measures and other DBS-derived neurological biomarkers, and performed sensitivity analyses to assess the robustness of the observed associations.

## 2 Materials and methods

### 2.1 Study design and participants

This study was an analysis of de-identified data, conducted in accordance with the Declaration of Helsinki and the SHARE-European Research Infrastructure Consortium (SHARE-ERIC) Conditions of Use. We performed a cross-sectional analysis using data from the DBS biomarker substudy of SHARE (Börsch-Supan et al., 2013; Bergmann et al., 2019; Börsch-Supan et al., 2024; SHARE-ERIC, 2024; SHARE-ERIC, 2026). The p-tau217 substudy was restricted to residual DBS specimens from Germany, Sweden, and Denmark. These three countries therefore defined the source population for the present biomarker analysis. This study was reported in accordance with the STROBE guideline for observational studies. Participants were eligible if they were aged 50 years or older and had available DBS data, sufficient information to calculate AIP, measured p-tau217, and available DBS assay batch and fieldwork variables. The main analysis used a complete-case approach and required complete data for all covariates included in Model 5. Participants were excluded if TG or HDL-C were missing or non-positive, or if TG levels were ≥500 mg/dL. The final analytic sample included 2,676 participants.

### 2.2 DBS collection and biomarker assays

Non-fasting capillary blood was collected by finger prick onto dried blood spot (DBS) cards during SHARE Wave 6 in 2015, mailed to the SHARE Biobank, and stored at −23 °C. Remaining DBS material from Denmark, Germany, and Sweden was analyzed in 2025 at Statens Serum Institut, Copenhagen, Denmark. Using two 3.2-mm DBS punches, p-tau217 was measured with the S-PLEX Human Tau (pT217) Kit (K151APFS), whereas total tau, GFAP, and NfL were measured with the S-PLEX Neurology Panel 1 (K15639S; Meso Scale Diagnostics). Concentrations were expressed in fg/mL. Detailed assay performance characteristics are provided in Supplementary Table S1. The released Set 3 biomarker values were unadjusted DBS-derived concentrations; assay batch and DBS collection and transport variables were therefore included as covariates (Börsch-Supan et al., 2026).

Within-study analytical characterization showed that 14 of 2,676 p-tau217 measurements (0.5%) were below the laboratory-defined lower limit of detection. The intra-assay and inter-assay coefficients of variation for internal controls were 22.8 and 19.3%, respectively (Supplementary Table S1). These metrics provide study-specific information on detectability and internal analytical performance. Paired plasma or CSF p-tau217 measurements were not available in SHARE; therefore, the analyses focused on relative between-participant differences in continuous DBS-derived p-tau217.

### 2.3 Exposure

The primary exposure was AIP, calculated as log10(TG/HDL-C), with TG and HDL-C expressed in mmol/L. Values in mg/dL were converted to mmol/L by dividing TG by 88.57 and HDL-C by 38.67. In the main analyses, AIP was standardized by the standard deviation (SD) of the complete-case sample when modeled as a continuous variable. For quartile analyses, participants were classified into four groups using quartile cut points from the same complete-case sample.

For cross-metric comparisons, AIP, TG, HDL-C, non-HDL-C, TC/HDL-C, and Friedewald-estimated LDL-C were standardized and entered separately into the fully adjusted model. Because the Friedewald equation is not applicable at TG levels ≥400 mg/dL (Friedewald et al., 1972), all lipid-metric comparison models were fitted in the same analytic sample restricted to participants with TG < 400 mg/dL and complete data for all lipid metrics and covariates.

### 2.4 Outcomes

The primary outcome was p-tau217, with total tau, GFAP, and NfL evaluated as secondary outcomes. The LLODs were 93.86, 24.23, 84.82, and 147.40 fg/mL, respectively. Values below the LLOD occurred in 14 of 2,676 p-tau217 measurements (0.5%), none of 2,825 total tau measurements, 43 of 2,825 GFAP measurements (1.5%), and 565 of 2,825 NfL measurements (20.0%). These values were replaced with LLOD/√2 (Succop et al., 2004), after which biomarker concentrations were natural log-transformed and standardized within the corresponding analytic sample.

### 2.5 Covariates

Covariates were entered using prespecified nested models. Model 1 adjusted for age, sex, and country. Model 2 additionally adjusted for DBS assay batch and fieldwork variables. Model 3 further included years of education, body mass index (BMI), smoking status, alcohol consumption, and physical activity. Model 4 additionally adjusted for self-reported medical conditions, including diabetes, hypertension, high cholesterol, heart disease, stroke, and chronic kidney disease (CKD). Model 5 additionally adjusted for lipid-lowering medication use and served as the main analytic model. Covariate selection and the nested model structure were informed by a conceptual directed acyclic graph (Supplementary Figure S1).

DBS fieldwork variables included transport time, drying time, sampling temperature, whether the storage bag had been opened, desiccant status, and blood spot size. Country and DBS assay batch were treated as categorical variables.

### 2.6 Statistical analysis

Baseline characteristics were described across quartiles of AIP. Continuous variables are presented as means (SDs) or medians (interquartile ranges), and categorical variables as counts (percentages). Tests for trend were performed using linear regression or logistic regression, as appropriate. Biomarkers with skewed distributions were natural log-transformed before descriptive analyses and model fitting.

Associations between AIP and biomarker outcomes were estimated using multivariable linear regression with heteroskedasticity-consistent type 3 (HC3) robust standard errors. In the main analysis, z[ln(p-tau217)] was modeled as the outcome and z(AIP) as the exposure. Results are reported as β coefficients, 95% confidence intervals (CIs), and *p* values. To facilitate interpretation of effect magnitude, the standardized coefficient was converted to the original ln(p-tau217) scale using the sample standard deviation of ln(p-tau217), and the resulting estimate was exponentiated to express the corresponding percentage difference in geometric mean p-tau217 concentration. Dose–response patterns were assessed using AIP quartiles and restricted cubic splines. The spline model used knots at the 5th, 35th, 65th, and 95th percentiles of AIP, and tests were performed for the overall association and for departure from linearity.

For analyses involving Friedewald-estimated LDL-C, participants with TG ≥ 400 mg/dL were excluded. Comparisons of the six lipid metrics with p-tau217 were performed in a common sample with complete data for all lipid metrics, p-tau217, and Model 5 covariates (*n* = 2,582). The lipid metrics and ln(p-tau217) were standardized within this sample, and model fit was compared using AIC, BIC, and ΔR^2^. Cross-biomarker analyses were further restricted to participants with complete data for p-tau217, total tau, GFAP, and NfL (*n* = 2,580). Within this sample, all lipid metrics and biomarker outcomes were standardized, Model 5 was fitted for each of the 24 lipid metric–biomarker pairs, and the Benjamini-Hochberg false discovery rate (FDR) correction was applied across these associations. Results are presented as standardized β coefficients.

All analyses were conducted using R version 4.5.2. Two-sided *p* values <0.05 were considered statistically significant.

### 2.7 Sensitivity analyses

Sensitivity analyses included excluding participants using lipid-lowering medications; excluding participants with diabetes, myocardial infarction/heart disease, stroke, or CKD; excluding those with self-reported dementia at baseline; winsorizing AIP and ln(p-tau217) at the 1st and 99th percentiles; further adjusting for the European Depression scale (EURO-D) score; and repeating the main analysis within each country.

Potential selection bias was assessed using two-stage inverse-probability weighting (IPW) in the age-eligible three-country source population (*N* = 8,109). Stage-specific stabilized weights for p-tau217 availability and complete-case inclusion were multiplied, truncated at the 1st and 99th percentiles, and applied to Model 5.

To assess potential overadjustment, we repeated Model 5 without self-reported high cholesterol, using the same analytic sample and all other covariates. We also calculated variance inflation factors (VIFs) for AIP and self-reported high cholesterol in the full model. To assess whether the results depended on the functional form of age, we repeated Model 5 with age modeled using a restricted cubic spline with knots at the 5th, 35th, 65th, and 95th percentiles. All analyses were cross-sectional association analyses; no temporal ordering or causal pathway was evaluated.

## 3 Results

### 3.1 Study population and baseline characteristics

Among 24,218 records in the SHARE Wave 6 DBS biomarker module, 23,996 participants were aged ≥50 years. Of these, 8,109 were from Germany, Sweden, or Denmark, the three countries included in the p-tau217 substudy, and 2,908 had available p-tau217 measurements. Within this p-tau217 sample, 48 participants were excluded because of missing, invalid, or non-positive TG or HDL-C values, 40 because TG was ≥500 mg/dL, and 144 because of incomplete Model 5 covariates, yielding a final analytic sample of 2,676 participants (Figure 1). Characteristics of participants included in and not included in the primary analytic sample are presented in Supplementary Table S2. Among the 2,820 participants assessed for Model 5 completeness, 144 (5.1%) lacked at least one covariate, most commonly years of education (3.6%), BMI (1.1%), or smoking status (0.7%). Missingness was 0.1% for lipid-lowering medication use, <0.1% for each of the six self-reported medical conditions, and absent for all other covariates; categories were not mutually exclusive. Across AIP quartiles, higher AIP was accompanied by a lower proportion of women, lower educational attainment, higher BMI, and higher prevalence of diabetes, hypertension, high cholesterol, stroke, chronic kidney disease, and lipid-lowering medication use (Table 1).

**Figure 1:**
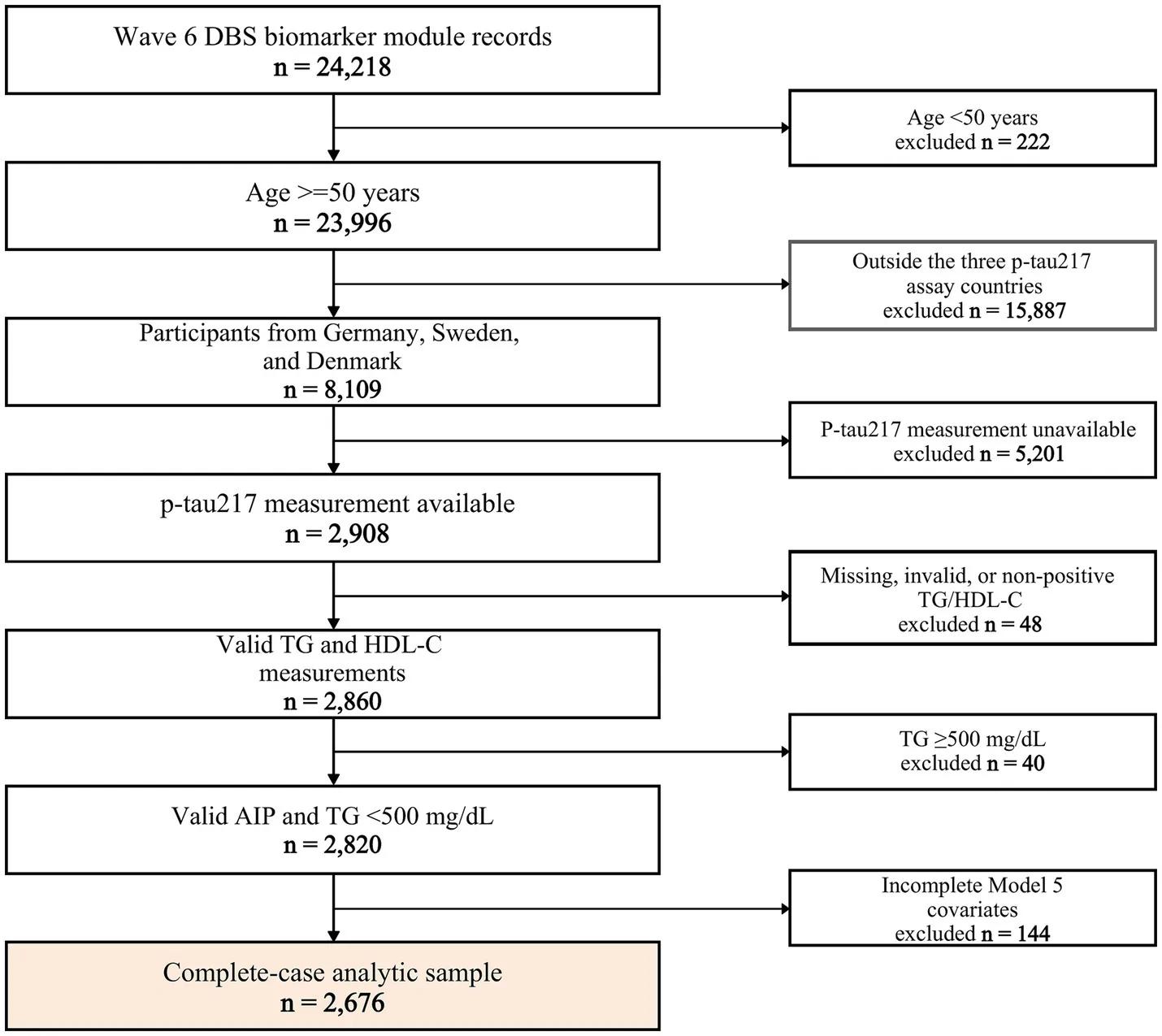
Flow diagram of participant selection. Flow diagram showing the selection of the complete-case analytic sample from the SHARE Wave 6 DBS biomarker module. The final complete-case analytic sample included 2,676 participants. AIP, atherogenic index of plasma; DBS, dried blood spot; HDL-C, high-density lipoprotein cholesterol; LLOD, lower limit of detection; p-tau217, phosphorylated tau 217; SHARE, Survey of Health, Ageing and Retirement in Europe; TG, triglycerides.

**Table 1:** Baseline characteristics according to quartiles of the atherogenic index of plasma.

Characteristic	Q1 (*n* = 669)	Q2 (*n* = 669)	Q3 (*n* = 669)	Q4 (*n* = 669)	*p* value for trend
Age, years, mean (SD)	66.1 (9.1)	66.2 (9.5)	66.3 (9.2)	66.0 (8.9)	0.968
Female, *n* (%)	414 (61.9%)	386 (57.7%)	358 (53.5%)	332 (49.6%)	<0.001
Education, years, mean (SD)	13.1 (3.7)	13.1 (3.8)	12.8 (3.9)	12.6 (3.7)	0.005
BMI, kg/m^2^, mean (SD)	24.9 (4.1)	26.2 (4.4)	26.7 (4.6)	28.1 (4.8)	<0.001
Ever smoked daily, *n* (%)	370 (55.3%)	396 (59.2%)	363 (54.3%)	393 (58.7%)	0.530
Alcohol use in past 7 days, *n* (%)	505 (75.5%)	505 (75.5%)	485 (72.5%)	479 (71.6%)	0.054
Physical inactivity, *n* (%)	17 (2.5%)	25 (3.7%)	33 (4.9%)	31 (4.6%)	0.027
Diabetes, *n* (%)	37 (5.5%)	53 (7.9%)	74 (11.1%)	100 (14.9%)	<0.001
Hypertension, *n* (%)	210 (31.4%)	241 (36.0%)	257 (38.4%)	307 (45.9%)	<0.001
High cholesterol, *n* (%)	127 (19.0%)	129 (19.3%)	152 (22.7%)	162 (24.2%)	0.007
Heart disease, *n* (%)	48 (7.2%)	55 (8.2%)	70 (10.5%)	64 (9.6%)	0.056
Stroke, *n* (%)	16 (2.4%)	10 (1.5%)	15 (2.2%)	32 (4.8%)	0.005
Chronic kidney disease, *n* (%)	6 (0.9%)	3 (0.4%)	4 (0.6%)	14 (2.1%)	0.034
Lipid-lowering medication, *n* (%)	131 (19.6%)	137 (20.5%)	146 (21.8%)	172 (25.7%)	0.006
HbA1c, %, mean (SD)	5.8 (0.4)	5.9 (0.4)	5.9 (0.5)	6.0 (0.6)	<0.001
ln(CRP), median (IQR)	0.63 (0.35, 1.02)	0.76 (0.45, 1.13)	0.85 (0.49, 1.27)	0.92 (0.56, 1.34)	<0.001
ln(cystatin C), median (IQR)	−0.05 (−0.11, 0.01)	−0.03 (−0.09, 0.04)	−0.02 (−0.08, 0.05)	0.00 (−0.06, 0.08)	<0.001
AIP, mean (SD)	−0.17 (0.10)	0.01 (0.04)	0.14 (0.04)	0.32 (0.08)	<0.001
ln(p-tau217), median (IQR)	7.49 (7.11, 7.86)	7.42 (7.04, 7.79)	7.52 (7.09, 7.87)	7.57 (7.20, 7.90)	0.002
ln(total tau), median (IQR)	9.15 (8.92, 9.40)	9.19 (8.94, 9.44)	9.21 (8.94, 9.45)	9.30 (9.03, 9.55)	<0.001
ln(GFAP), median (IQR)	7.03 (6.54, 7.40)	6.94 (6.47, 7.40)	6.94 (6.50, 7.40)	6.93 (6.45, 7.37)	0.030
ln(NfL), median (IQR)	7.55 (6.27, 8.28)	7.40 (5.84, 8.27)	7.65 (6.14, 8.36)	7.49 (6.18, 8.34)	0.504

Data are presented as mean (SD), median (IQR), or *n* (%), as appropriate. AIP quartiles were defined as Q1, AIP ≤ − 0.054; Q2, −0.054 < AIP ≤0.067; Q3, 0.067 < AIP ≤0.205; and Q4, AIP >0.205. Cut points are rounded to three decimal places. Biomarker values are natural log-transformed. AIP was calculated as log10(triglycerides/high-density lipoprotein cholesterol). p values for trend were calculated by modeling AIP quartile as an ordinal variable using linear regression for continuous variables and logistic regression for binary variables. Two-sided *p* values < 0.05 were considered statistically significant. BMI, body mass index; CRP, C-reactive protein; GFAP, glial fibrillary acidic protein; HbA1c, glycated hemoglobin; IQR, interquartile range; NfL, neurofilament light chain; p-tau217, phosphorylated tau 217; SD, standard deviation.

### 3.2 AIP was positively associated with DBS-derived p-tau217

In sequentially adjusted models, higher AIP was consistently associated with higher ln-transformed p-tau217. In the fully adjusted model, a 1-SD higher AIP, corresponding to 0.192 AIP units, was associated with a 0.074-SD higher ln-transformed p-tau217 level (95% CI, 0.034–0.115; *p* < 0.001). After back-transformation, this corresponded to a 5.2% higher adjusted geometric mean DBS-derived p-tau217 concentration (95% CI, 2.3–8.1%; Figure 2A).

**Figure 2:**
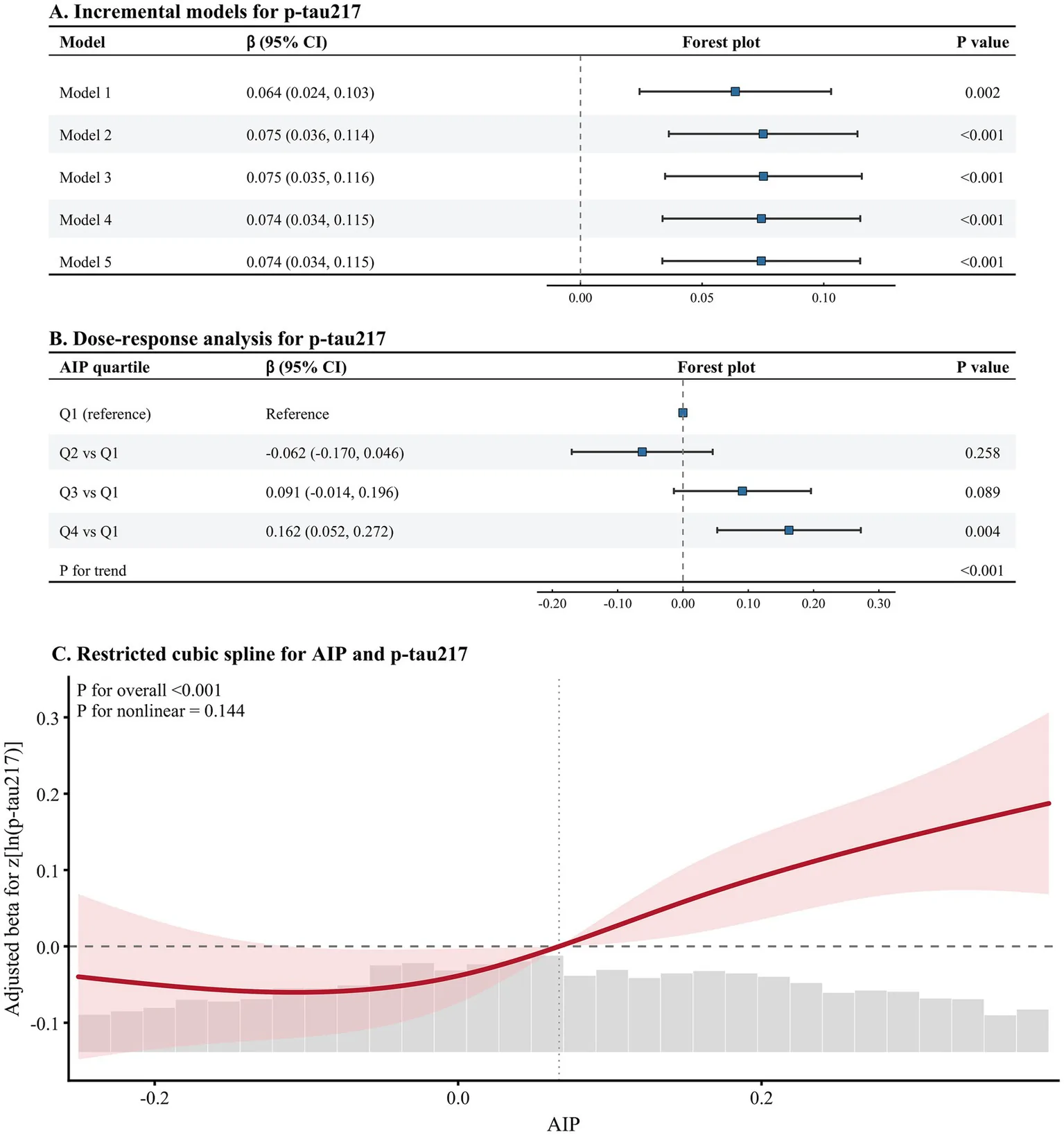
Association of AIP with DBS-derived p-tau217 across incremental models and dose–response analyses. Association between AIP and standardized natural log-transformed p-tau217 [z(ln[p-tau217])]. **(A)** Associations across incremental multivariable models. **(B)** Dose–response association by AIP quartiles, with the lowest quartile as the reference group. **(C)** Restricted cubic spline analysis of AIP and z(ln[p-tau217]), displayed from the 5th to the 95th percentile of AIP. The solid line represents the adjusted difference in z(ln[p-tau217]) relative to the median AIP, indicated by the vertical dotted line. The shaded area represents the 95% confidence interval, and the histogram shows the distribution of AIP within the plotted range. *p* overall tests the overall association, whereas *p* for departure from linearity compares the spline model with the linear model. Effect estimates are *β* coefficients with 95% confidence intervals, representing the difference in z(ln[p-tau217]) per 1 SD increase in AIP or relative to the reference quartile. AIP, atherogenic index of plasma; CI, confidence interval; p-tau217, phosphorylated tau 217.

Estimates across AIP quartiles were not strictly monotonic. Relative to Q1, the estimate was −0.062 for Q2 (95% CI, −0.170 to 0.046; *p* = 0.258), 0.091 for Q3 (95% CI, −0.014 to 0.196; *p* = 0.089), and 0.162 for Q4 (95% CI, 0.052 to 0.272; *p* = 0.004). The ordinal trend test was statistically significant (*p* for trend < 0.001), although the individual quartile estimates showed variation (Figure 2B). The restricted cubic spline analysis showed an overall association (*p* overall < 0.001), with the curve appearing relatively flat or slightly declining at lower AIP values and rising more clearly at higher values. The test for departure from linearity was not statistically significant (*p* = 0.144), leaving the precise dose–response shape uncertain (Figure 2C).

### 3.3 Associations of AIP across neurological biomarkers

In the common lipid-metric comparison sample (*n* = 2,582), AIP remained associated with higher p-tau217 (β, 0.075; 95% CI, 0.036–0.115; *p* < 0.001). The AIP model had the lowest AIC and BIC values (7245.0 and 7409.0, respectively; Figure 3A). Higher TG, non-HDL-C, and TC/HDL-C values and lower HDL-C were also associated with higher p-tau217. Friedewald-estimated LDL-C was not associated with p-tau217 (β, 0.001; 95% CI, −0.041–0.043; *p* = 0.969). The LDL-C model had higher AIC and BIC values (7258.8 and 7422.7, respectively).

**Figure 3:**
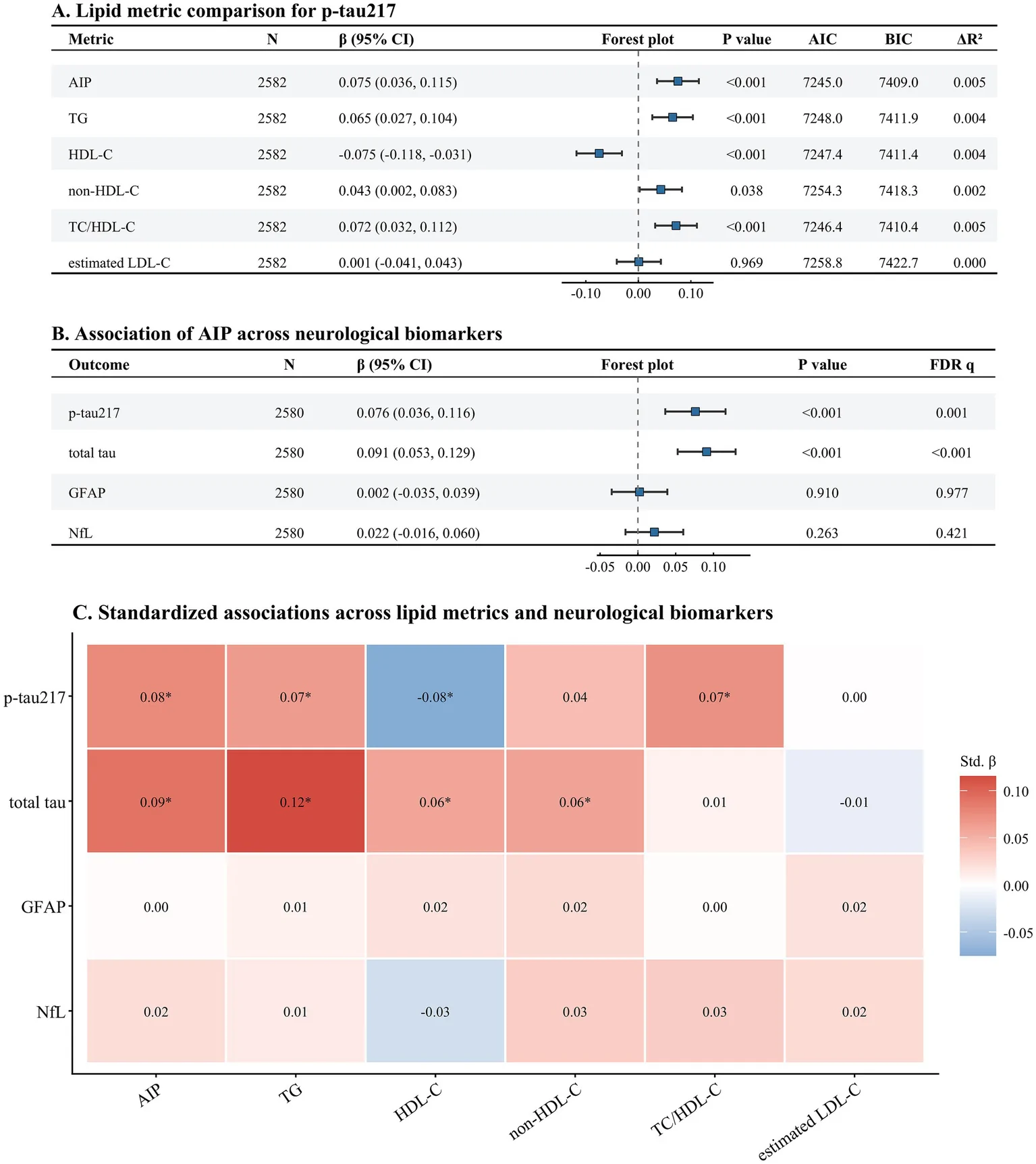
Comparative analyses of lipid metrics and neurodegeneration-related blood biomarkers. **(A)** AIP, TG, HDL-C, non-HDL-C, TC/HDL-C ratio, and Friedewald-estimated LDL-C as standardized predictors of *z*(*ln*[p-tau217]) in separate fully adjusted models, with model fit summarized by AIC, BIC, and Δ*R*^2^. All models were fitted in the same analytic sample with TG < 400 mg/dL and complete data for all lipid metrics and covariates (*n* = 2,582). **(B)** Cross-biomarker comparison of AIP associations with p-tau217, total tau, GFAP, and NfL, with false discovery rate correction. **(C)** Heatmap of standardized *β* coefficients across lipid metrics and neurobiomarker outcomes; asterisks indicate statistically significant associations after multiple-testing correction. Panels B and C were based on the same common analytic sample with complete data for all six lipid metrics, all four neurological biomarkers, and Model 5 covariates, restricted to participants with TG < 400 mg/dL (*n* = 2,580). FDR correction was applied across all 24 lipid metric–biomarker associations. AIC, Akaike information criterion; AIP, atherogenic index of plasma; BIC, Bayesian information criterion; CI, confidence interval; FDR, false discovery rate; GFAP, glial fibrillary acidic protein; HDL-C, high-density lipoprotein cholesterol; LDL-C, low-density lipoprotein cholesterol; NfL, neurofilament light chain; TC, total cholesterol; TG, triglycerides.

For the cross-biomarker comparison, analyses were restricted to the common comparison sample (*n* = 2,580). AIP was positively associated with both p-tau217 (standardized β, 0.076; 95% CI, 0.036–0.116; FDR *q* = 0.001) and total tau (standardized β, 0.091; 95% CI, 0.053–0.129; FDR *q* < 0.001), whereas its associations with GFAP and NfL were not statistically supported after FDR correction. The standardized point estimates for AIP, TG, and non-HDL-C were numerically larger for total tau than for p-tau217; however, no formal between-outcome comparisons were performed. The cross-biomarker patterns were not identical: HDL-C was inversely associated with p-tau217 but positively associated with total tau, whereas TC/HDL-C was associated with p-tau217 but not with total tau. Full results are presented in Figures 3B,C and Supplementary Table S4.

### 3.4 Sensitivity analyses

The primary Model 5 association was statistically significant (*p* < 0.001) and remained significant after exclusion of lipid-lowering medication users (β, 0.071; 95% CI, 0.024–0.118; *p* = 0.003), exclusion of participants with major cardiometabolic disease (β, 0.075; 95% CI, 0.029–0.121; *p* = 0.001), exclusion of self-reported dementia (β, 0.074; 95% CI, 0.033–0.115; *p* < 0.001), winsorization at the 1st and 99th percentiles (β, 0.075; 95% CI, 0.036–0.115; *p* < 0.001), and additional adjustment for EURO-D (β, 0.076; 95% CI, 0.035–0.117; *p* < 0.001; Supplementary Figure S2A). Country-stratified analyses yielded positive estimates in Denmark (β, 0.068; 95% CI, 0.007–0.129; *p* = 0.0295) and Germany (β, 0.098; 95% CI, 0.021–0.174; *p* = 0.0125), with a directionally positive but less precise estimate in Sweden (β, 0.075; 95% CI, −0.008–0.158; *p* = 0.0788; Supplementary Figure S2B).

Excluding self-reported high cholesterol produced a nearly identical estimate (β, 0.075; 95% CI, 0.034–0.115; *p* < 0.001; primary model: β, 0.074). In the full model, the VIFs were 1.13 for AIP and 2.28 for self-reported high cholesterol, suggesting no substantial multicollinearity. Modeling age with a restricted cubic spline yielded an essentially identical estimate (β, 0.074; 95% CI, 0.034–0.115; *p* < 0.001). Two-stage IPW improved covariate balance (maximum absolute SMD, 0.258 before vs. 0.011 after weighting). The weighted estimate remained essentially unchanged (β, 0.075; 95% CI, 0.033–0.117; *p* < 0.001; Supplementary Table S3).

## 4 Discussion

In this cross-sectional analysis of SHARE data, higher AIP was associated with higher DBS-derived p-tau217 concentrations among adults aged 50 years or older. After multivariable adjustment, the association was statistically significant but modest in magnitude (β per 1-SD higher AIP, 0.074; 95% CI, 0.034–0.115; *p* < 0.001). A 1-SD higher AIP was associated with a 5.2% higher adjusted geometric mean DBS-derived p-tau217 concentration (95% CI, 2.3–8.1%). Dose–response analyses suggested an overall positive association that was most apparent at higher AIP values, particularly in the highest quartile (Q4 vs. Q1: β, 0.162; 95% CI, 0.052–0.272; *p* = 0.004), but the quartile estimates were not strictly monotonic. Findings were consistent across sensitivity analyses. By examining AIP in relation to DBS-derived p-tau217, this study extends prior evidence linking AIP to vascular risk, cognitive performance, and incident dementia. But the biological and clinical significance of this modest cross-sectional association remains to be established.

Evidence on blood lipids in relation to cognition and dementia remains mixed. The associations of TC, LDL-C, HDL-C, and TG may vary in direction across age periods, follow-up durations, and study populations. For example, there is relatively consistent evidence linking elevated LDL-C in midlife to a higher long-term risk of dementia (Iwagami et al., 2021; Anstey et al., 2008). In later life, however, the relationship between a single LDL-C measurement and cognitive outcomes is often more complex, and may be influenced by survival bias, prodromal changes in body weight and metabolism, lipid-lowering treatment, and reverse causation (Livingston et al., 2024; Iwagami et al., 2021; Af Geijerstam et al., 2025). In our study, Friedewald-estimated LDL-C showed no clear association with p-tau217, whereas AIP, TG, HDL-C, and TC/HDL-C displayed more consistent association patterns. This finding suggests that, in older populations, LDL-C level alone may be insufficient to capture lipid metabolic abnormalities relevant to brain aging. By contrast, AIP, which reflects the combined phenotype of elevated TG and reduced HDL-C, may more closely represent insulin resistance, residual atherogenic risk, and the burden of triglyceride-rich lipoproteins. It may therefore better capture lipid abnormalities associated with AD-related blood biomarkers than LDL-C alone.

AIP was associated with both p-tau217 and total tau in the common comparison sample. Although the standardized estimate was numerically larger for total tau, this does not imply greater value as an AD biomarker, because the analysis did not assess diagnostic performance or pathological specificity, nor did it formally compare the two estimates. p-tau217 is closely linked to AD-related amyloid and tau pathology (Ashton et al., 2024), whereas conventional blood total tau is less pathologically specific and may predominantly reflect peripheral rather than brain-derived tau (Gonzalez-Ortiz et al., 2023). Both p-tau217 and total tau were associated with AIP and TG, suggesting some overlap in their metabolic correlates. Yet their relationships with HDL-C and TC/HDL-C differed, indicating that the two tau measures may not convey the same biological information. AIP showed no clear association with GFAP or NfL after FDR correction. Because GFAP reflects astrocytic activation linked partly to amyloid-related processes, whereas NfL reflects neuroaxonal injury across a range of neurological conditions (Pereira et al., 2021; Gaetani et al., 2019), the overall findings indicate a non-uniform biomarker pattern rather than a generalized association across neurological biomarkers. Multimodal studies incorporating amyloid PET, tau PET, and CSF biomarkers are needed to clarify the biological basis of these differences.

Potential explanations for the observed cross-sectional association have been proposed in prior vascular and neurodegeneration literature, including vascular injury, blood–brain barrier dysfunction, insulin resistance, and inflammation. Elevated AIP generally indicates higher TG, lower HDL-C, and a more atherogenic lipoprotein profile. This state often coexists with insulin resistance, endothelial dysfunction, chronic low-grade inflammation, and small-vessel injury (Mechanick et al., 2020; Dobiásová and Frohlich, 2001; Onat et al., 2010). Chen et al. provided experimental evidence that HPDA/Zn-mediated CREB inhibition reduced markers of inflammation and plaque vulnerability and improved atherosclerotic plaque stability (Chen et al., 2023). Atherosclerosis and cerebral small-vessel disease may, in turn, reduce cerebrovascular reserve and increase white matter injury and microvascular ischemia, potentially weakening neuronal resilience to amyloid-β (Aβ)- and tau-related stress (Snyder et al., 2015). Disruption of the blood–brain barrier may allow peripheral inflammatory mediators, coagulation-related factors, and lipid-associated signals to enter the central nervous system, with potential effects on neuroinflammation, Aβ clearance, and tau phosphorylation (Nation et al., 2019; Sweeney et al., 2018). Systemic metabolic disturbances may influence AD-related processes through insulin resistance, metabolic inflammation, and glial nutrient sensing, with potential effects on neuroinflammation, oxidative stress, energy metabolism, and phagocytic clearance (Wei et al., 2021; Yan et al., 2026). Microglia-mediated inflammation also appears modifiable (Guo et al., 2025). Within this inflammatory framework, BDNF–TrkB signaling may function as a protective pathway at the interface between vascular and neural injury. Activation of BDNF–TrkB signaling attenuated neuroinflammation and improved cognitive deficits (Luo et al., 2019; Rana et al., 2025). Collectively, these studies provide a biological rationale for further investigating systemic inflammation within the broader vascular-metabolic context of the observed association between AIP and p-tau217.

This study has several strengths. First, it was based on a well-characterized three-country subsample of the SHARE aging cohort. Second, the analyses accounted for DBS-related technical factors and field collection variables, partly addressing measured pre-analytical variability. Third, we examined the dose–response relationship, compared AIP with conventional lipid measures, and compared association patterns across multiple DBS-derived neurological biomarkers. Fourth, sensitivity analyses addressed several potential sources of bias, including lipid-lowering medication use, major cardiometabolic diseases, self-reported dementia, extreme values, depressive symptoms, and between-country differences.

Several limitations should be considered. First, AIP and p-tau217 were measured contemporaneously; therefore, the cross-sectional design precludes establishing temporal sequence or causal direction, including whether higher AIP preceded or caused higher p-tau217. Second, p-tau217 measurements were available only for a subset of participants from three countries, potentially limiting generalizability and introducing selection bias. Although IPW substantially improved covariate balance and yielded a similar estimate, residual bias from unmeasured selection factors remains possible. Third, single-time-point measurements could not capture long-term lipid exposure or trajectories of biomarker change. Fourth, the study lacked amyloid PET, tau PET, paired plasma or CSF biomarker measurements, and follow-up clinical outcomes, limiting assessment of the biological and clinical significance of the observed association. Fifth, DBS-derived p-tau217 was measured in specimens collected in 2015, stored at −23 °C, and analyzed approximately 10 years later. Validation under identical assay and storage conditions remains limited. Thus, these data do not establish equivalence with plasma or CSF p-tau217 in biological interpretation, numerical values, or diagnostic performance. Sixth, 20.0% of NfL measurements were below the LLOD and were replaced with LLOD/√2; this left-censoring may have attenuated the estimated NfL association and limits interpretation of this secondary outcome.

In summary, this study found that higher AIP was associated with higher DBS-derived blood p-tau217 levels, and this association remained robust after extensive adjustment and across multiple sensitivity analyses. The combined TG and HDL-C pattern captured by AIP may reflect a broader cardiometabolic milieu associated with higher p-tau217 levels. Several interconnected biological processes, including vascular injury, metabolic dysfunction, blood–brain barrier impairment, and low-grade inflammation, may be relevant to the observed association. Future longitudinal and multimodal studies are needed to clarify the temporal direction, pathological specificity, and biological significance of this association.

## 5 Conclusion

In this cross-sectional analysis, higher AIP, reflecting atherogenic dyslipidemia, was associated with a modestly higher DBS-derived p-tau217 concentration in older adults. The biological and clinical significance of this association remains to be established in longitudinal and multimodal studies.

## Data Availability

The data used in this study are available from the Survey of Health, Ageing and Retirement in Europe (SHARE) Research Data Center. Access to SHARE data is granted to registered users for scientific research after agreement to the SHARE Conditions of Use. The SHARE Dried Blood Spot data analyzed in this study are available from SHARE-ERIC, Release 2.0.0, under the persistent identifier https://doi.org/10.6103/SHARE.w6.DBS.200. Because these are third-party controlled-access data, the authors are not permitted to redistribute the individual-level data. Researchers may apply for access directly through SHARE at https://share-eric.eu/data/. The analysis code is available from the corresponding author upon reasonable request, subject to compliance with the SHARE Conditions of Use.
