# Supplementary material for: Association between atherogenic dyslipidemia and DBS-derived p-tau217 in older adults: evidence from SHARE DBS data
Source: Front Aging Neurosci. 2026 Sep 22;18:1948518. doi: 10.3389/fnagi.2026.1948518 (PMC13638366; doi:10.3389/fnagi.2026.1948518)
Supplement: Supplementary file 1 [file Data_Sheet_1.PDF]

Supplementary Material

1 Supplementary tables

Supplementary Table 1. Analytical characteristics of the SHARE Set 3 neurodegeneration biomarkers.

Biomarker	Assay (catalog no.)	LLOD (fg/mL)	ULOD (fg/mL)	Intra-assay CV (%)	Inter-assay CV (%)	Below LLOD in analytic sample
p-tau217	S-PLEX Human Tau (pT217), K151APFS	93.86	500,000	22.8	19.3	14/2,676 (0.5%)
Total tau	S-PLEX Neurology Panel 1, K15639S	24.23	100,000	7.91	8.73	0/2,825 (0%)
GFAP	S-PLEX Neurology Panel 1, K15639S	84.82	557,500	15.0	13.4	43/2,825 (1.5%)
NfL	S-PLEX Neurology Panel 1, K15639S	147.40	2,831,250	18.7	14.3	565/2,825 (20.0%)

Note: Assays were performed at Statens Serum Institut, Copenhagen, Denmark, using the Meso Scale Diagnostics S-PLEX platform. Intra-assay CVs were based on 37 replicate internal-control measurements, and inter-assay CVs were based on duplicate controls analyzed across 45 plates. Values below the LLOD were replaced with LLOD/$\sqrt{2}$ before natural log transformation. CV, coefficient of variation; GFAP, glial fibrillary acidic protein; LLOD, lower limit of detection; NfL, neurofilament light chain; p-tau217, phosphorylated tau 217; ULOD, upper limit of detection.

Supplementary Table 2. Characteristics of participants included in and not included in the primary analytic sample.

Characteristic	Included (n=2,676)	Not included (n=5,433)	Absolute SMD
Age, years	66.1 (9.2); n=2,676	67.5 (9.4); n=5,433	0.150
Female sex	1,490/2,676 (55.7%)	2,899/5,433 (53.4%)	0.047
Country: Germany	707/2,676 (26.4%)	2,147/5,433 (39.5%)	0.281
Country: Sweden	748/2,676 (28.0%)	1,822/5,433 (33.5%)	0.121
Country: Denmark	1,221/2,676 (45.6%)	1,464/5,433 (26.9%)	0.396
Education, years	12.9 (3.8); n=2,676	12.6 (3.7); n=5,135	0.087
Body mass index, kg/m²	26.5 (4.6); n=2,676	26.6 (4.5); n=5,346	0.029
Atherogenic index of plasma	0.071 (0.192); n=2,676	0.077 (0.201); n=2,429	0.029
Ever smoked daily	1,522/2,676 (56.9%)	2,823/5,377 (52.5%)	0.088
Alcohol use in the past 7 days	1,974/2,676 (73.8%)	3,729/5,433 (68.6%)	0.113
Physical inactivity	106/2,676 (4.0%)	264/5,430 (4.9%)	0.044
Diabetes	264/2,676 (9.9%)	668/5,431 (12.3%)	0.078
Hypertension	1,015/2,676 (37.9%)	2,163/5,431 (39.8%)	0.039
High cholesterol	570/2,676 (21.3%)	1,096/5,431 (20.2%)	0.028
Heart disease	237/2,676 (8.9%)	556/5,431 (10.2%)	0.047
Stroke	73/2,676 (2.7%)	197/5,431 (3.6%)	0.051
Chronic kidney disease	27/2,676 (1.0%)	82/5,431 (1.5%)	0.045
Lipid-lowering medication	586/2,676 (21.9%)	1,180/5,424 (21.8%)	0.003

Note: Values are mean (SD); n available for continuous variables and n/N (%) for categorical variables. Included participants were members of the fully adjusted Model 5 complete-case analytic sample. The source population (N=8,109) comprised SHARE Wave 6 dried blood spot participants aged ≥50 years from Germany, Sweden, and Denmark; all other source-population participants were classified as not included. The comparison was restricted to these three biomarker-assay countries to distinguish within-substudy selection from country eligibility. AIP was summarized only when triglyceride and HDL cholesterol values were positive and triglycerides were <500 mg/dL, consistent with the analytic eligibility criteria. SMDs are absolute values calculated from available observations; country categories were evaluated as separate binary indicators. Larger SMDs indicate greater imbalance. No hypothesis tests were performed because the comparison was descriptive. AIP, atherogenic index of plasma; BMI, body mass index; HDL, high-density lipoprotein; SMD, standardized mean difference.

Supplementary Table 3. Diagnostics and results of the two-stage inverse-probability-weighted sensitivity analysis.

Panel A. Sample and weight diagnostics

Diagnostic	Value
Stage 1 source population, n	8,109
Stage 1 with p-tau217 available, n	2,908
Stage 2 complete-case analytic sample, n	2,676
Untruncated stabilized weights, range	0.618 to 2.171
Truncated stabilized weights, range	0.638 to 1.689
Effective sample size after weighting	2,472

Panel B. Covariate balance

Characteristic	Absolute SMD before weighting	Absolute SMD after weighting
Age, years	0.101	0.002
Female sex	0.031	0.011
Country: Germany	0.191	<0.001
Country: Sweden	0.082	<0.001
Country: Denmark	0.258	<0.001
Education, years	0.055	0.007
Body mass index, kg/m²	0.018	0.002
Ever smoked daily	0.052	0.007
Alcohol use in the past 7 days	0.077	0.002
Physical inactivity	0.030	0.004
Diabetes	0.053	0.005
Hypertension	0.026	0.004
High cholesterol	0.019	0.003
Heart disease	0.032	<0.001
Stroke	0.035	0.008
Chronic kidney disease	0.031	0.002
Lipid-lowering medication	0.003	0.002
Maximum absolute SMD	0.258	0.011

Panel C. Association between AIP and ln(p-tau217)

Analysis	β	95% CI	P value
Primary complete-case Model 5	0.074	0.034 to 0.115	<0.001
Two-stage IPW Model 5	0.075	0.033 to 0.117	<0.001

Note: The source population comprised 8,109 SHARE Wave 6 DBS participants aged ≥50 years from Germany, Sweden, and Denmark. Two logistic selection models were fitted. Stage 1 estimated the probability of p-tau217 availability in the source population using the covariates listed in Panel B. Stage 2 estimated the probability of inclusion in the final analytic sample among participants with available p-tau217, using the same covariates plus ln(p-tau217). Final inclusion required calculable AIP, TG <500 mg/dL, and complete Model 5 covariates. For the selection models, missing continuous covariates were replaced with source-population medians and binary covariates with

modes; covariate-specific missingness indicators were included in Stage 1 only. These replacements were used only to estimate selection probabilities. At each stage, the stabilized numerator was the observed marginal selection probability, and the denominator was the individual probability predicted by the corresponding logistic model. The two stage-specific weights were multiplied and truncated by winsorization at the 1st and 99th percentiles. Absolute SMDs compare the analytic sample with the source population before and after weighting. The weighted outcome model retained the complete Model 5 covariate set and used HC3 robust standard errors. AIP and ln(p-tau217) were standardized using the means and SDs of the primary analytic sample; β represents the change in standardized ln(p-tau217) per 1-SD higher AIP. ESS was calculated as $(\Sigma w)^2/\Sigma w^2$, where w denotes the truncated stabilized weight.

Abbreviations: AIP, atherogenic index of plasma; CI, confidence interval; DBS, dried blood spot; ESS, effective sample size; HC3, heteroskedasticity-consistent type 3; IPW, inverse probability weighting; ln, natural logarithm; p-tau217, phosphorylated tau 217; SMD, standardized mean difference; TG, triglycerides.

Supplementary Table 4. Standardized associations of lipid metrics with neurological biomarkers in the common analytic sample.

Biomarker	Lipid metric	n	Standardized β (95% CI)	P value	FDR q value
p-tau217	AIP	2,580	0.076 (0.036, 0.116)	<0.001	0.001
	TG	2,580	0.066 (0.027, 0.104)	<0.001	0.003
	HDL-C	2,580	-0.075 (-0.118, -0.032)	<0.001	0.003
	non-HDL-C	2,580	0.043 (0.002, 0.083)	0.038	0.100
	TC/HDL-C	2,580	0.072 (0.032, 0.112)	<0.001	0.002
	estimated LDL-C	2,580	0.001 (-0.041, 0.043)	0.980	0.980
Total tau	AIP	2,580	0.091 (0.053, 0.129)	<0.001	<0.001
	TG	2,580	0.115 (0.077, 0.154)	<0.001	<0.001
	HDL-C	2,580	0.059 (0.016, 0.102)	0.008	0.023
	non-HDL-C	2,580	0.060 (0.020, 0.099)	0.003	0.010
	TC/HDL-C	2,580	0.008 (-0.032, 0.049)	0.694	0.793
	estimated LDL-C	2,580	-0.014 (-0.056, 0.027)	0.492	0.622
GFAP	AIP	2,580	0.002 (-0.035, 0.039)	0.910	0.977
	TG	2,580	0.009 (-0.027, 0.045)	0.636	0.764
	HDL-C	2,580	0.019 (-0.021, 0.059)	0.356	0.503
	non-HDL-C	2,580	0.024 (-0.015, 0.063)	0.228	0.405
	TC/HDL-C	2,580	0.002 (-0.037, 0.040)	0.937	0.977
	estimated LDL-C	2,580	0.019 (-0.019, 0.056)	0.329	0.493
NfL	AIP	2,580	0.022 (-0.016, 0.060)	0.263	0.421
	TG	2,580	0.014 (-0.024, 0.052)	0.470	0.622

Biomarker	Lipid metric	n	Standardized β (95% CI)	P value	FDR q value
	HDL-C	2,580	-0.029 (-0.072, 0.013)	0.177	0.353
	non-HDL-C	2,580	0.032 (-0.006, 0.070)	0.098	0.215
	TC/HDL-C	2,580	0.033 (-0.006, 0.071)	0.094	0.215
	estimated LDL-C	2,580	0.023 (-0.015, 0.062)	0.236	0.405

Note: Fully adjusted linear regression models with HC3 robust standard errors were fitted separately for each lipid metric–biomarker pair in the same analytic sample with complete data for all six lipid metrics, all four neurological biomarkers, and Model 5 covariates. Because LDL-C was estimated using the Friedewald equation, the common sample was restricted to participants with TG <400 mg/dL (n=2,580). All lipid metrics and natural log-transformed biomarkers were standardized within this common sample. P values were adjusted across the 24 associations using the Benjamini–Hochberg false discovery rate procedure. β values represent the difference in standardized ln-transformed biomarker concentration per 1-SD higher lipid metric. AIP, atherogenic index of plasma; CI, confidence interval; FDR, false discovery rate; GFAP, glial fibrillary acidic protein; HDL-C, high-density lipoprotein cholesterol; LDL-C, low-density lipoprotein cholesterol; NfL, neurofilament light chain; TC, total cholesterol; TG, triglycerides.

2 Supplementary figures

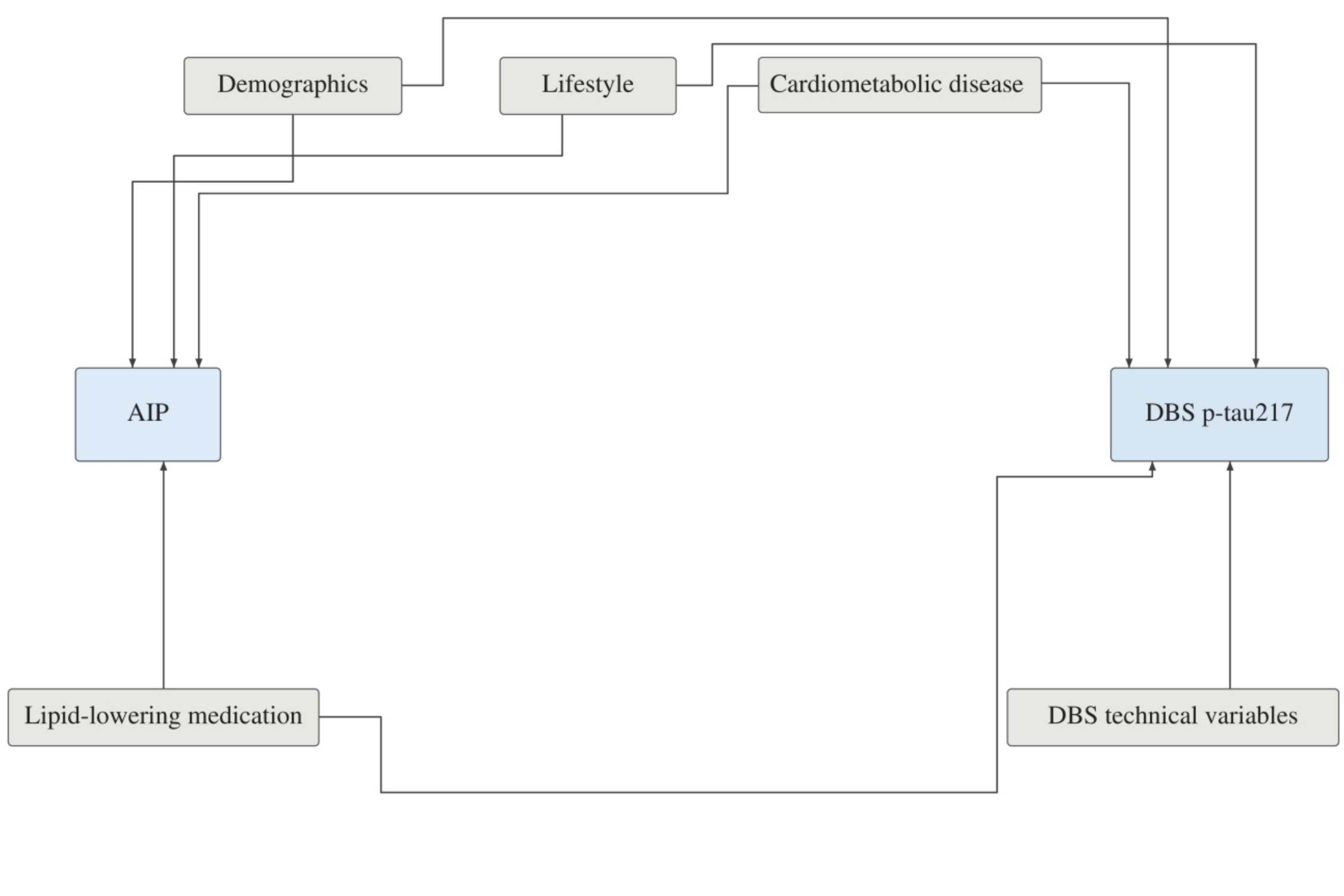

Supplementary Figure 1. Conceptual directed acyclic graph for the association between AIP and DBS-derived blood p-tau217. The diagram illustrates the assumed relationships among the atherogenic index of plasma (AIP), dried blood spot (DBS)-derived blood p-tau217, and adjustment

domains. Demographic factors, lifestyle-related factors, cardiometabolic comorbidities, lipid-lowering medication use, and DBS technical variables were considered potential confounders or technical sources of variation. Arrows indicate assumed conceptual relationships used to guide model specification and do not establish causal effects. AIP, atherogenic index of plasma; DBS, dried blood spot; p-tau217, phosphorylated tau 217.

A. Sensitivity analyses

Analysis	N	β (95% CI)	Forest plot	P value
Primary Model 5	2676	0.074 (0.034, 0.115)		<0.001
Age modeled with restricted cubic spline	2676	0.074 (0.034, 0.115)		<0.001
Excluding lipid-lowering medication users	2090	0.071 (0.024, 0.118)		0.003
Excluding major cardiometabolic disease	2156	0.075 (0.029, 0.121)		0.001
Excluding self-reported dementia	2652	0.074 (0.033, 0.115)		<0.001
Winsorized at 1st/99th percentiles	2676	0.075 (0.036, 0.115)		<0.001
Additional adjustment for EURO-D	2664	0.076 (0.035, 0.117)		<0.001

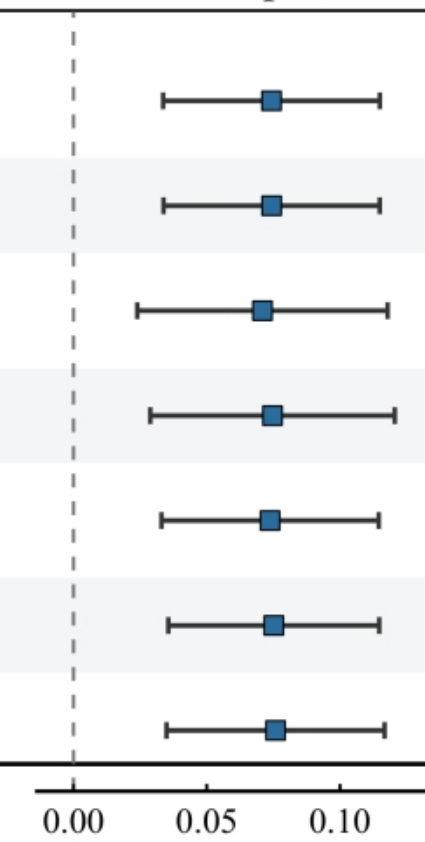

B. Country-stratified analyses

Country	N	β (95% CI)	Forest plot	P value
Denmark	1221	0.068 (0.007, 0.129)		0.0295
Germany	707	0.098 (0.021, 0.174)		0.0125
Sweden	748	0.075 (-0.008, 0.158)		0.0788

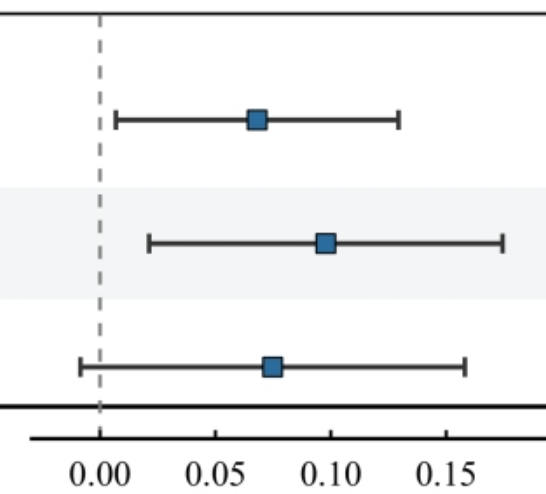

Supplementary Figure 2. Sensitivity and country-stratified analyses of the association between AIP and blood p-tau217. (A) Results from the primary fully adjusted model and sensitivity analyses. Separate sensitivity analyses modeled age using a restricted cubic spline with knots at the 5th, 35th, 65th, and 95th percentiles; excluded lipid-lowering medication users, participants with major cardiometabolic disease (diabetes, myocardial infarction/heart disease, stroke, or CKD), or participants with self-reported dementia; winsorized AIP and ln(p-tau217) at the 1st and 99th percentiles; or additionally adjusted for the EURO-D depressive symptom score. (B) Country-stratified estimates for Denmark, Germany, and Sweden. Effect estimates are β coefficients and 95% confidence intervals per 1-SD increase in AIP. AIP, atherogenic index of plasma; CI, confidence interval; CKD, chronic kidney disease; EURO-D, European Depression scale; p-tau217, phosphorylated tau 217.
